# Understanding Moment‐to‐Moment Processing of Visual Narratives

**DOI:** 10.1111/cogs.12699

**Published:** 2018-11-16

**Authors:** John P. Hutson, Joseph P. Magliano, Lester C. Loschky

**Affiliations:** ^1^ Department of Psychology The College of Wooster; ^2^ Department of Learning Georgia State University; ^3^ Department of Psychological Sciences Kansas State University

**Keywords:** Eye tracking, Eye movements, Scene perception, Narrative comprehension, Visual attention, Bridging inference generation, Picture stories

## Abstract

What role do moment‐to‐moment comprehension processes play in visual attentional selection in picture stories? The current work uniquely tested the role of bridging inference generation processes on eye movements while participants viewed picture stories. Specific components of the Scene Perception and Event Comprehension Theory (SPECT) were tested. Bridging inference generation was induced by manipulating the presence of highly inferable actions embedded in picture stories. When inferable actions are missing, participants have increased viewing times for the immediately following critical image (Magliano, Larson, Higgs, & Loschky, 2016). This study used eye‐tracking to test competing hypotheses about the increased viewing time: (a) *Computational Load*: inference generation processes increase overall computational load, producing longer fixation durations; (b) *Visual Search*: inference generation processes guide eye‐movements to pick up inference‐relevant information, producing more fixations. Participants had similar fixation durations, but they made more fixations while generating inferences, with that process starting from the fifth fixation. A follow‐up hypothesis predicted that when generating inferences, participants fixate scene regions important for generating the inference. A separate group of participants rated the inferential‐relevance of regions in the critical images, and results showed that these inferentially relevant regions predicted differences in other viewers’ eye movements. Thus, viewers’ event models in working memory affect visual attentional selection while viewing visual narratives.

## 1 Introduction

**Figure 1:**
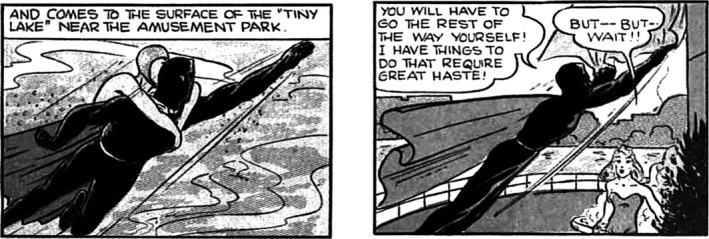
Panels from Fantoman (Gustavson, 1939). Panel 1 shows Fantoman diving up through the water of a lake with a woman hanging on his neck. Panel 2 shows Fantoman starting to fly off, with the woman now standing on the ground next to the lake.

In any narrative, it is impossible to convey all the events that make it up. This is just as true for visual narratives (e.g., comics, childrens picture stories; Cohn, 2013; Magliano, Kopp, Higgs, & Rapp, 2016; Magliano, Larson, Higgs, & Loschky, 2016) as it is for linguistically conveyed narratives (oral or written) (Clark, 1977). Take, for example, the two panels below from Fantoman (Gustavson, 1939) shown in Fig. 1. The first panel shows Fantoman diving up through the water of a lake with a woman hanging onto his neck. The second panel shows Fantoman and the woman on the ground, of what looks like the shore of a lake, with the woman no longer clinging on to his neck, and he starting to fly off into the sky. How are we to interpret the gap between these panels? There is a break in space, time, and actions, which affects how narrative events are processed (Zwaan, Magliano, & Graesser, 1995; Zwaan & Radvansky, 1998). In order to maintain narrative coherence, readers should interpret the second panel as a continuation of the story from the first panel (e.g., Graesser, Singer, & Trabasso, 1994; Zwaan & Radvansky, 1998). However, inferring that the second panel is a continuation of the events potentially requires one to infer events that fill the gap between those explicitly depicted in the panels, such as the characters having exited the water, flown to the shore, landed, etc.

Inferences that connect story elements are called *bridging inferences*. Virtually all theories of narrative comprehension assume bridging inferences are critical for constructing a coherent event model for a narrative (McNamara & Magliano, 2009). One type of bridging inference involves inferring events that causally link explicit content (e.g., Clark, 1977; Magliano, Kopp, et al., 2016; Magliano, Larson, et al., 2016; Singer & Halldorson, 1996), which is illustrated in Fig. 1. While there is some debate as to what aspects of comprehension are modality independent (e.g., Loughlin, Grossnickle, Dinsmore, & Alexander, 2015), there is little doubt that bridging inferences are important for comprehension regardless of the modality of a narrative (Magliano, Higgs, & Clinton, in press; Magliano, Loschky, Clinton, & Larson, 2013; Magliano, Larson, et al., 2016).

However, it is less obvious whether the cognitive systems that support inference generation are similar across modalities (Loughlin et al., 2015; Magliano, Kopp, et al., 2016; Magliano, Larson, et al., 2016). Theories of narrative comprehension have traditionally emphasized the role of processes that are either constructive (e.g., causal reasoning; Graesser et al., 1994; Trabasso, van den Broek, & Suh, 1989) or memory‐based (Kintsch, 1988, 1998; Singer & Halldorson, 1996). There are good reasons to believe that these cognitive systems are enlisted to support the comprehension of graphic narratives (and film) as well (Gernsbacher, Varner, & Faust, 1990; Kintsch, 1998). In fact, recent EEG work with sequential narratives has shown effects consistent with inference generation results in both updating the narrative mental model and activating information in long‐term memory (Cohn & Kutas, 2015). However, it is also possible that processes that support the understanding of complex visual scenes, and specifically those that support scene perception (Henderson & Hollingworth, 1999), are also enlisted to support bridging inferences in graphic narratives (Magliano, Larson, et al., 2016). This study explores this possibility.

**Figure 2:**
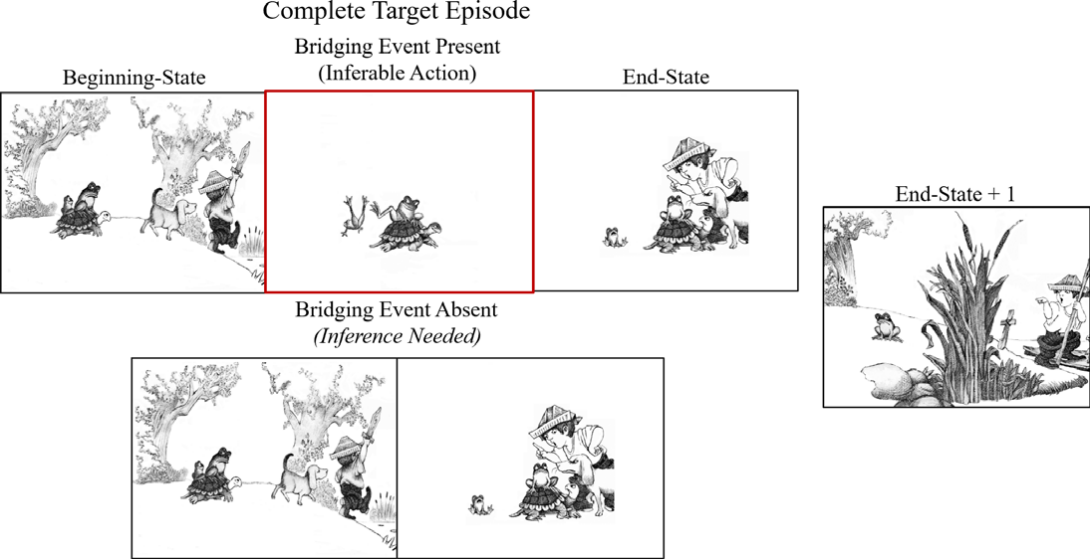
Example target episode with bridging event‐present and ‐absent illustrated. The missing bridging event, that the big frog “kicked off” the little frog from the turtle's back, would need to be generated in event‐absent trials on the end‐state image. The end‐state + 1 image is the image immediately following the end‐state image in both the bridging event‐present and ‐absent conditions.

The current study builds on previous work reported in Magliano, Larson, et al. (2016). The study also investigated the processes involved in generating bridging inferences in visual narratives. Participants viewed wordless picture stories that contained target events that consisted of three panels: beginning state, bridging event, and end‐state (see Fig. 2). The authors manipulated whether the bridging event picture was present in the target sequences. Interestingly, in a think‐aloud task, they found evidence consistent with their prediction that participants were more likely to spontaneously mention the bridging event when the corresponding panel was *absent* than when it was present (59% of the time compared to 42%); likely due to the inference being more highly activated in working memory than for the actually physically present and perceived action—an example of the generation effect (Bertsch, Pesta, Wiscott, & McDaniel, 2007; Slamecka & Graf, 1978). Importantly, in a follow‐up silent viewing experiment, they found that viewing times on the end‐state picture were longer when the bridging event was absent than when it was present. The think‐aloud and viewing time data converged to suggest that participants inferred the bridging events when their absence created a break in the narratives coherence (see also Cohn & Kutas, 2015; Cohn & Wittenberg, 2015), as is the case when reading texts that contain missing events (e.g., Clark, 1977; Singer & Halldorson, 1996). Magliano, Kopp, et al. (2016) found similar effects of missing bridging events on picture viewing times, but also showed that inferring bridging events distorts memory for the visual content. That is, participants tended to falsely recognize the missing bridging event pictures as having been seen when they later took a recognition memory task in which they are asked to identify which pictures had been shown in the stories. This result is consistent with false recognition effects for inferred information in text (Bransford & Johnson, 1973; Johnson, Bransford, & Solomon, 1973; Kintsch, Welsch, Schmalhofer, & Zimny, 1990), and scenes (Hannigan & Reinitz, 2001, 2003).

The increased viewing times found when viewers generate bridging inferences in visual narratives raise an important question, namely, what is their functional significance? There are at least two possibilities, although they are not mutually exclusive.

One possibility is that the increased viewing times are due to making longer fixation durations which represent increased computational load. In studies of eye movements in reading, comprehension difficulties at the discourse level result in longer first fixations and gaze durations (Just & Carpenter, 1980). For example, when readers of a text must search their working memory (WM) representation for the antecedent to a pronoun (Ehrlich & Rayner, 1983), or an atypical noun (Duffy & Rayner, 1990), or compare their current understanding of a sentence with their WM representation of a previously seen picture (Underwood, Jebbett, & Roberts, 2004), they produce longer fixation or gaze durations. Even more to the point, when readers are prompted to generate an elaborative inference, but the text provides insufficient constraint (i.e., weak context and an implicit referent), it also produces longer first fixations and gaze durations (O'Brien, Shank, Myers, & Rayner, 1988). Importantly, work on the effect of working memory and computational load have also been extended beyond reading text. Eye movement studies that directly manipulated cognitive load (e.g., number of items in WM to search for (Gould, 1973)) show similar increases in fixation durations with an increase in load (Gould, 1973; Zingale & Kowler, 1987). Other research has shown that when reading single panel cartoons (e.g., “The Far Side”) containing a caption and an image, fixation durations on the image were longer when it was seen after the caption than before the caption (Carroll, Young, & Guertin, 1992). These longer fixation durations were explained as being due to the reader needing to integrate the mental representation of the caption, in WM, with the representation of the currently viewed image, also in WM, in order to comprehend the joke (assumedly at the level of the event model). An important caveat to consider is that stimulus properties also have a strong effect on fixation durations (e.g., images that have been degraded tend to have longer fixation durations) (Loftus, 1985; Loftus, Kaufman, Nishimoto, & Ruthruff, 1992; Loschky, McConkie, Yang, & Miller, 2005; Mannan, Ruddock, & Wooding, 1995). As such, in this study, all participants viewed the same images; thus, stimulus effects should be controlled for. In addition to this eye movement work, the EEG study mentioned above (Cohn & Kutas, 2015) indicates that similar processes of updating event model information in working memory and reactivating information in working memory occur when reading text and picture stories. Thus, based on these previous eye movement studies of reading and scene perception and EEG studies of picture stories, we might predict that the longer viewing times produced when viewers are generating bridging inferences in visual narratives are due to increased computational load in WM, which would be evidenced by longer first fixations or gaze durations.

An alternative possibility is that viewers produce longer viewing times due to making more fixations in the scene while searching for cues from the picture content to either *facilitate generating* the bridging inference, or to *validate* it after generating it. This would be similar to the same domain‐general cognitive process of integrating information into the event model. However, modality‐specific processes would change how such information is activated in working memory for integration into the event model. Specifically, due to high visual resolution being limited to the fovea (Loschky et al., 2005; Wilkinson, Anderson, Bradley, & Thibos, 2016), gathering new information from the scene often requires making additional saccades to new scene areas. We can think of this as a case of a somewhat inefficient visual search for relevant information, which will produce longer total viewing times due to making additional overt shifts of visual attention, and thus more eye fixations (DeAngelus & Pelz, 2009; Howard, Gilchrist, Troscianko, Behera, & Hogg, 2011; Wolfe, Vo, Evans, & Greene, 2011; Yarbus, 1967). This is likely somewhat analogous to what happens in reading when the inference generation process runs into trouble, such as the gender of a pronoun mismatching that of its referent (Ehrlich & Rayner, 1983), or a protagonist's action either violating a predictive inference (Calvo, Meseguer, & Carreiras, 2001) or violating a previously inferred goal for the protagonist (Poynor & Morris, 2003). In all such cases, readers typically make regressive eye movements immediately after encountering the unexpected word, in an attempt to reanalyze the preceding context to reduce the perceived incoherence. Similarly, the above cited study of single panel comic reading (Carroll et al., 1992) also found that when a previously read caption, whose representation was in WM did not match the currently viewed image, readers made 33% more fixations in order to determine the mismatch.

Based on the above, if viewers are found to *only* make longer fixations, it would be consistent with the idea that the bridging inference process causes a cognitive load during inference generation but does not require searching for cues to facilitate that process or to validate it. Conversely, if viewers are found to *only* make more fixations, it would be consistent with the idea that generating the bridging inference requires visual search for cues to generate or validate the inference but does not incur a cognitive load during the inference generation process. Of course, these alternative hypotheses are not logically mutually exclusive. Thus, if viewers are found to make *both* longer fixations *and* more fixations, it would be consistent with both the cognitive load and visual search hypotheses.

In attempting to understand how processes that support scene perception may also support bridging inferences, we use the explanatory framework of the Scene Perception and Event Comprehension Theory (SPECT) (Loschky, Hutson, Smith, Smith, & Magliano, 2018). SPECT is an integrative framework, which synthesizes several theories from the areas of scene perception (e.g., Henderson & Hollingworth, 1999; Irwin, 1996; Oliva, 2005), event perception (e.g., Kurby & Zacks, 2008; Zacks, Speer, & Reynolds, 2009), and narrative text comprehension (Gernsbacher, 1990; Zwaan & Radvansky, 1998). It starts with perceptual processing during single eye fixations and extends to processes that support the construction of a coherent event model, such as inference generation. SPECT distinguishes between front‐end and back‐end processes. Front‐end processes are involved in information extraction. They comprise a set of processes involving gist processing (Loschky & Larson, 2010; Oliva, 2005), object recognition (Cichy, Khosla, Pantazis, Torralba, & Oliva, 2016), and motion detection (implied or real, Osaka, Matsuyoshi, Ikeda, & Osaka, 2010). Additionally, front‐end processes involve attentional‐selection, which is affected by both endogenous and exogenous factors when viewing complex scenes (Findlay & Walker, 1999; Henderson, 2007; Itti & Borji, 2015; Wolfe, 2007). Front‐end processes occur during single eye fixations, specifically information extraction from a scene during a fixation, and attentional selection during a fixation, which determines where the eyes will go for the next fixation. In this study, we are particularly interested in the front‐end process of attentional selection, as indicated by fixation locations (Corbetta et al., 1998; Deubel & Schneider, 1996).

Back‐end processes occur in memory (either working memory [WM] or long‐term memory [LTM]) to integrate information across multiple eye fixations (Irwin, 1996; Irwin & Gordon, 1998; Pertzov, Avidan, & Zohary, 2009). In this study, we are particularly interested in the processes involved in creating the current event model in WM, namely the mental representation of what is occurring now (Radvansky & Zacks, 2014). SPECT argues for three main processes in the creation of the event model: (a) laying the foundation for a new event model, (b) mapping in‐coming information to the current event model, and (c) shifting to create a new event model (Gernsbacher, 1990, 1997). When a new event model must be started, the viewer must understand the most basic facts of the new event, such as the spatio‐temporal framework (where and when the event takes place), the major entities (who is involved) and key objects, and the actions/events (what is happening) (Zwaan & Radvansky, 1998). For example, in Panel 1 of Fig. 1, Fantoman and a woman (major entities) are diving up (event) through the water of a lake (location).

In this study, we are particularly interested in the process of mapping in‐coming information to the current event model, because this is when it is argued that bridging inferences are needed. One instance when this occurs is when new information comes in during a new fixation, such as when a viewer first fixates Panel 2 of Fig. 1 that shows Fantoman has placed the woman he was saving on the ground next to the water they were in and is flying away. During this first fixation of Panel 2, the viewer must determine whether there is a new event, or whether the new information is a continuation of the current event, in which case, the new information should be mapped onto the current event model. This requires assessing the coherence of the new information with the current event model (Gernsbacher, 1997). Some cases are more obviously coherent than others. In the case of Panel 2 (Fig. 1), which has a different spatial location from Panel 1 (Under water compared to above water), the question is whether a bridging inference is sufficient to maintain coherence with the current event model (Graesser et al., 1994). If so, knowledge of the world, from LTM, is used to fill‐in gaps in the event model during the mapping stage (Graesser et al., 1994). However, when a gap is detected, are attentional selection systems engaged to find content in the image that helps infer the events that causally bridge those explicitly shown? In this study, we aimed to answer this question.

### 1.1 Study overview

This study was designed to test novel hypotheses generated by SPECT concerning the relationship between back‐end and front‐end processes. The visual search hypothesis states that the back‐end process of mapping in‐coming information to the current event model can influence the front‐end process of attentional selection. More specifically, when a break in narrative coherence is identified, the back‐end process of generating a bridging inference can influence front‐end attentional selection, which results in making more fixations to pick up information necessary for making the inference. Note that this is a novel and detailed hypothesis for a very specific type of top–down control of attention (i.e., inference generation processes guiding attentional selection). The alternative hypothesis is the computational load hypothesis as described above—namely, generating inferences consumes working memory resources and thus leads to longer fixation durations. Furthermore, the alternative computational load hypothesis, that the event model affects processing time, but not attentional selection, is supported by what we know about the time courses of bridging inference generation and attentional selection.

We know that the back‐end process of bridging inference generation takes about 400 ms processing time when reading text (Magliano, Baggett, Johnson, & Graesser, 1993) and picture stories (Cohn & Kutas, 2015), which is longer than attentional selection requires. Specifically, attentional selection is finished by about 80 ms before the end of an eye fixation (Caspi, Beutter, & Eckstein, 2004). An eye fixation in viewing scenes is on average 330 ms (Rayner, 1998), so in scenes attentional selection takes about 250 ms. When reading text, the average eye fixation is 250 ms (Rayner, 1998), so in text attentional selection takes about 170 ms. Indeed, most studies of the effects of inference generation on eye movements during text reading have shown delayed effects that occur after fixating the inferential target, either during later rereading of the target or while fixating later words in the text, assumedly during an integration stage (Calvo, 2001; Calvo et al., 2001; Myers, Cook, Kambe, Mason, & O'Brien, 2000; O'Brien et al., 1988; Poynor & Morris, 2003). Therefore, the increased viewing time may be more likely to manifest as longer fixation durations due to the increased computational load in WM of inference generation. Of course, as noted above, the computational load and visual search hypotheses need not be mutually exclusive, so it is possible that we could find evidence consistent with both (i.e., longer fixation durations and more fixations).

Note that this type of top–down attentional selection, if it exists, would be an example of what has been called a *mandatory* top–down influence on attentional selection (Baluch & Itti, 2011) in that the effect occurs through an automated process typically outside of the viewer's conscious awareness. This is as opposed to a *volitional* top–down process. Our own prior studies have shown only weak effects of the viewer's event model on their attentional selection in the domain of viewing film clips (Hutson, Smith, Magliano, & Loschky, 2017; Loschky, Larson, Magliano, & Smith, 2015). Thus, our goal in this study is to better understand the processes involved in generating bridging inferences in visual narratives involving eye movements and, in doing so, test a novel hypothesis from SPECT.

## 2 Experiment 1: Eye‐tracking

### 2.1 Method

#### 2.1.1 Participants

Seventy‐nine participants (58 women, 21 men) from the Kansas State University undergraduate participant pool participated in Experiment 1 for course credit and were included in analyses. Five participants were dropped for not having complete data due to issues with the eye‐tracking (e.g., difficulty calibrating resulting in the participant not having time to finish the experiment, or loss of track during the experiment resulting in the participant not having data for at least one full picture story). All participants in the experiment had normal or corrected‐to‐normal visual acuity (measured using the Freiburg Visual Acuity Test [FrACT]; Bach, 2006). The Kansas State University Institutional Review Board approved all experiments in the study. All participants completed the approved informed consent before participating in the experiment. Analyses were performed on de‐identified data.

#### 2.1.2 Materials

Six picture stories (ranging from 24–26 images each) from the Boy, Dog, Frog series were used (Mayer, 1967, 1973, 1974, 1975, 1980; Mayer & Mayer, 1971). Four target episodes were identified for each story, giving a total of 24 target episodes in the experiment. These target episodes, as illustrated in Fig. 2, consist of three‐image sequences: (a) a beginning‐state, (b) a bridging event, and (c) an end‐state. Importantly, as mentioned above, when the bridging event is absent, a think‐aloud procedure showed that participants readily infer the missing event on the end‐state image (Magliano, Larson, et al., 2016). Some modifications were made to the images from the original picture stories. Namely, the original stories often had a large amount of background detail that was removed for the experiment to put the focus of the materials on the characters and events in the narrative.

#### 2.1.3 Apparatus

Images were presented at a resolution of 1,024 × 768 on a 17″ ViewSonic Graphics Series CRT monitor (Model G90fb). A chin and forehead rest set a fixed viewing distance of 60.96 cm. The screen subtended 31.8° × 24.1° of visual angle.

Eye‐tracking was done using an Eyelink1000 eye tracker. Eye position was sampled 1,000 times per second (1,000 Hz). Participants started with a nine‐point calibration procedure to ensure an average spatial accuracy of 0.5° of visual angle and a maximum error of 1°. A chin and forehead rest were used to maintain an accurate track throughout the experiment. If participant calibration deviated from the specified spatial accuracy, recalibration was completed before they started the next picture story.

#### 2.1.4 Design

A within‐subjects design was used. In each story, for the four target episodes, participants saw two bridging event present episodes and two bridging event absent episodes. This was counterbalanced, such that if “bridging event present” were “A” and “bridging event absent” were “B,” there are six possible orderings: (1) AABB, (2) ABAB, (3) ABBA, (4) BBAA, (5) BABA, and (6) BAAB. These six bridging event presence orderings were combined with the six stories’ presentation orders in a 6 × 6 Latin square, resulting in 36 order combinations. Thus, for every 36 participants, each bridging event presence condition was presented an equal number of times in each possible ordering and equally across each story, with each story also presented equally often in each ordering.

For analyses, a second within‐subjects condition was also included. All participants saw the end‐state image of a target episode, and they also saw the image immediately after the end‐state image (end‐state + 1) (Fig. 2). To test that effects measured are due to the bridging inference generation process, it is expected the differences between the bridging event‐present and ‐absent conditions would be localized to the end‐state image, rather than spilling over to the end‐state + 1 image. Thus, the end‐state + 1 image served as a control for the end‐state image to measure inference generation processes. Overall, this resulted in a within‐subjects 2 (bridging event presence: present vs. absent) × 2 (picture: end‐state vs. end‐state + 1) design.

#### 2.1.5 Procedure

The procedure was the same used in Magliano, Larson, et al. (2016), except that eye‐tracking was added. Participants were instructed they would see six picture stories, and that after each one they would be asked to summarize the events of the narrative (in 3–4 sentences). This was done to motivate participants to comprehend the narrative of each story. The narrative summaries were not analyzed for this study. Narratives were presented with a single picture at a time, and participants progressed through them at their own pace by pressing a button labeled “Next” (the spacebar on a computer keyboard) to see the next picture in the story. At the beginning of each story, there was a drift check to ensure the eye‐tracker calibration was still good.

#### 2.1.6 Data processing

Both the viewing time and eye movement data were cleaned to remove outliers. The viewing time cleaning followed the same procedure as Magliano, Larson, et al. (2016). This criterion‐based cleaning set the minimum viewing time to 480 ms. This value was derived from the sum of (a) the average fixation duration length during typical scene viewing (330 ms; Henderson, 2007; Rayner, 1998), plus (b) the minimum time to make a simple manual reaction time response (150 ms; Teichner & Krebs, 1972). The maximum acceptable viewing time was set to 20 s. Magliano, Larson, et al. (2016) set this upper bound as it was approximately 3 standard deviations above the average viewing time, and viewing a single image in the picture for more than 20 s would indicate that a participant was likely not engaging in the comprehension task for the full duration they were viewing that image. This cleaning procedure resulted in 98 trials being removed from analyses (2.5% of trials).

The eye movement data were cleaned to remove outliers that were more likely to be due to tracker error (e.g., momentary loss of track) than to be a measure of a true eye movement behavior. The same dataset was used for analyses of both the fixation durations and number of fixations, to ensure any effects found were not due to the cleaning procedure. The cleaning removed the top and bottom 1% of fixation durations. The distribution of the data after cleaning had a minimum fixation duration of 60 ms and a maximum of 719 ms. More important, cleaning a fixation from the data also removed that fixation from the fixation count data for an image. As such, if there was an error in tracking during an image in the picture story when the data were cleaned, that trial would be removed from data analysis. Using this cleaning procedure, plus trials during which the eye tracking was totally lost (i.e., no data for the image in the picture story), a total of 184 trials were excluded from analyses (4.5% of trials).

All eye‐movement data were aggregated for each image. This was done to ensure the same number of observations for the fixation duration and number of fixations analyses. More specifically, the number of fixations on an image is necessarily a single number for that image. Thus, if the many fixation durations in a trial are not aggregated to also give a single mean fixation duration for each image, then comparisons between number of fixations and mean fixation durations would have more observations for the mean fixation durations, giving them more statistical power than the number of fixations. Nevertheless, the data were not aggregated across factors (i.e., Bridging event presence or Picture) in order to use image as an item random effect in the multilevel models. All the dependent variables analyzed had a strong positive skew, so they were log transformed for the analyses. Due to the different cleaning procedures between the viewing time and eye movement data, the degrees of freedom are not the same for the viewing time and eye movement analyses.

### 2.2 Results and discussion

All analyses used multilevel modeling. To keep the predictor variables consistent with Magliano, Larson, et al. (2016), bridging event presence and end‐state picture were the predictors. Since this was a repeated measures design, the random effect structure allowed both the participant and image intercepts to vary. Using the Akaike information criteria (AIC) (Burnham & Anderson, 2004), we determined that the participant by image (AIC = 3,908) random effects structure was superior to that for participant only (AIC = 4,784).

#### 2.2.1 Viewing time

**Figure 3:**
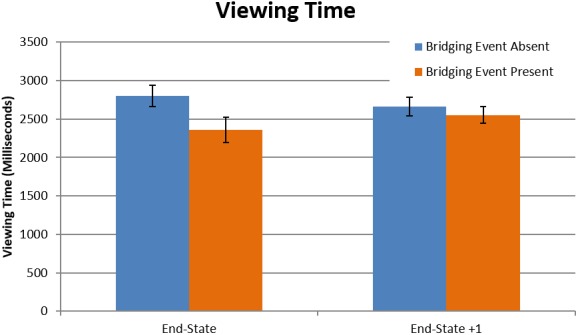
Mean viewing times as a function of bridging event presence and end‐state picture. Figure data were aggregated across event presence and end‐state picture for each participant. Results are presented in milliseconds (error bars are 1 *SE*).

The first analysis tested viewing time to replicate the results of Magliano, Larson, et al. (2016). As this was a self‐paced study, viewing time for an image was calculated as the time between consecutive pairs of participant button‐presses to progress from one image to the next image in the picture story. The previous viewing time results were indeed replicated (Table 1). The bridging event‐absent target episodes had longer viewing times (*M* =2,788 ms; *SD* = 1,901) compared to bridging event‐present episodes (*M* = 2,369 ms; *SD* = 1,498)^1^ (*t*(3567) = 6.57, *p* < .001), indicating that the inference generation process produced longer viewing times. Importantly, the interaction of the bridging event presence and picture was also significant (*t*(3567) = 4.43, *p* < .001), with the increase in viewing times for the bridging event‐absent condition localized to the end‐state image (Fig. 3).

**Table 1:** Summary of multilevel model for viewing time

	*B*	*SE(B)*	*t*	Sig. (*p*)
Intercept	7.70	.051	149.00	<.001
End‐state	−0.008	.033	−0.23	.817
Bridging event (absent)	0.04	.006	6.57	<.001
End‐state × Bridging event	0.03	.006	4.43	<.001

#### 2.2.2 Fixation durations versus number of fixations

Having replicated the viewing time results of Magliano, Larson, et al. (2016), we next tested the competing hypotheses concerning eye movements, specifically about which processes produced longer viewing times in the bridging event‐absent target episodes: computational load versus visual search. Fixation durations and number of fixations were calculated, using the default saccade velocity triggers of the SR Research eye‐movement data parser (EyeLink 1000 Plus User Manual Version 1.0.9, 2013). The parser identified a saccade when the eye was moving at a velocity ≥30° per second. If the eye was not identified as being in a saccade, it was recorded as being in fixation.

#### 2.2.3 Fixation durations

**Figure 4:**
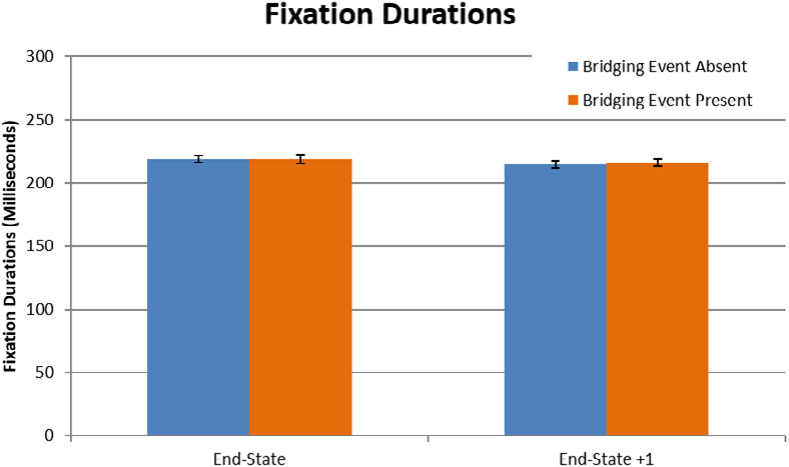
Mean fixation durations as a function of bridging event presence and end‐state picture. Figure data were aggregated across event presence and end‐state picture for each participant. Results are presented in milliseconds (error bars are 1 *SE*).

Participant fixation durations for the target episodes were used to test for support of the computational load hypothesis (Table 2). Overall, there was no effect of the bridging event presence on fixation durations (*t*(3484) = −.20, *p* = .841). The mean fixation duration for bridging event‐absent episodes was 218.5 ms (*SD* = 48), compared to a nearly identical 218.6 (*SD* = 50) for bridging event present (Fig. 4). Although there are no differences between the groups here, a very interesting and somewhat surprising point to note is that the fixation duration averages were closer to average fixation durations for reading (225–250 ms) (Rayner, 1998) than for viewing scenes (260–330 ms) (Henderson, 2007; Loschky & McConkie, 2002; Rayner, 2009). Thus, by this observation, the process of reading a picture story may share empirically grounded similarities with the process of reading text. When making this comparison between reading text and picture stories, it is important to consider factors of the medium that have previously been shown to influence fixation durations (e.g., the bottom‐up features of a scene). As is implied in the range of values for average fixation durations for reading text and scene viewing, factors other than the task (reading vs. scene viewing) also have a large influence on eye movements. As such, the low fixation durations in this study could be a result of the relatively sparse picture story drawings compared to photographs of scenes. However, the average fixation durations in scene perception (260–330 ms) are based on multiple studies and diverse stimulus sets. Since this study has an average fixation duration well below the lower end of the scene viewing fixation duration range, we speculate that the low fixation durations in this study are the result of the processes associated with the task of comprehending a visual narrative, which is the same task with reading text.

**Table 2:** Summary of multilevel model for fixation durations

	*B*	*SE(B)*	*t*	Sig. (*p*)
Intercept	5.36	.01	378.8	<.001
End‐state	0.007	.009	0.75	.456
Bridging event (absent)	−0.001	.003	−0.20	.841
End‐state × Bridging event	0.001	.003	0.40	.693

#### 2.2.4 Number of fixations

**Figure 5:**
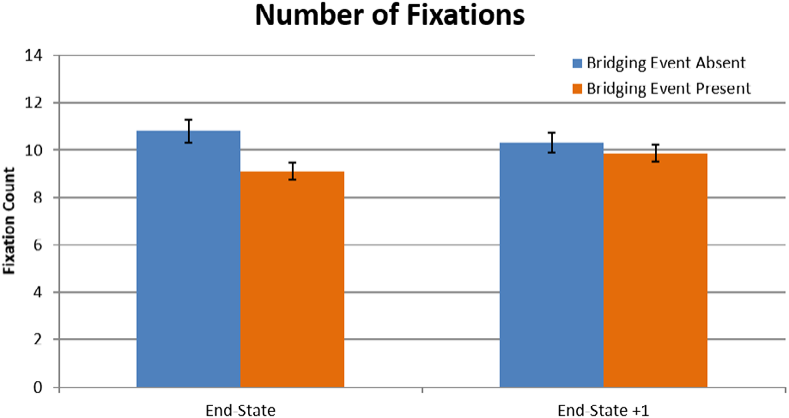
Mean number of fixations as a function of bridging event presence and end‐state picture (error bars are 1 *SE*). Figure data were aggregated across event presence and end‐state picture for each participant.

The numbers of fixations as a function of bridging event presence were used to test the visual search hypothesis (Table 3). Here, there was a significant effect of bridging event presence (*t*(3483) = 7.63, *p* < .001), with more fixations made in bridging event‐absent episodes (*M* = 10.8, *SD* = 6.6) than bridging event present episodes (*M* = 9.1, *SD* = 4.8) (Fig. 5). The effect was localized to the end‐state image, as seen through the significant interaction of bridging event presence and end‐state picture (*t*(3483) = 4.48, *p* < .001).

**Table 3:** Summary of multilevel model for number of fixations

	*B*	*SE(B)*	*t*	Sig. (*p*)
Intercept	2.17	.05	44.38	<.001
End‐state	−0.01	.033	−0.36	.718
Bridging event (absent)	0.05	.006	7.63	<.001
End‐state × Bridging event	0.03	.006	4.48	<.001

#### 2.2.5 Fixation count survival analysis

The above eye movement results show the Magliano, Larson, et al. (2016) viewing time effect was driven by an increase in the number of fixations participants made. A follow‐up question is at what fixation the divergence in the number of fixations begins. In other words, at what time point, as measured by fixations, does participant behavior in the event‐absent condition indicate that they have identified the break in narrative coherence? To address this question, a series of exploratory survival analyses were run on the eye movement data to further describe the fixation number effect (Carroll et al., 1992; Loschky & McConkie, 2005; Reingold & Sheridan, 2014, 2018; Yang & McConkie, 2001). Specifically, first an omnibus survival analysis was run on the fixation count data to verify that the event‐absent and ‐present groups did diverge, which should be the case given that mean difference shown above. Next, divergence point analyses (DPA) developed by Reingold and Sheridan (2014, 2018) were run to identify the ordinal fixation at which the divergence began. The divergence point analyses included an overall DPA, a confidence interval DPA that returns a divergence point with confidence intervals, and an individual participant DPA that calculates the divergence point from each participant's individual divergence points.

**Figure 6:**
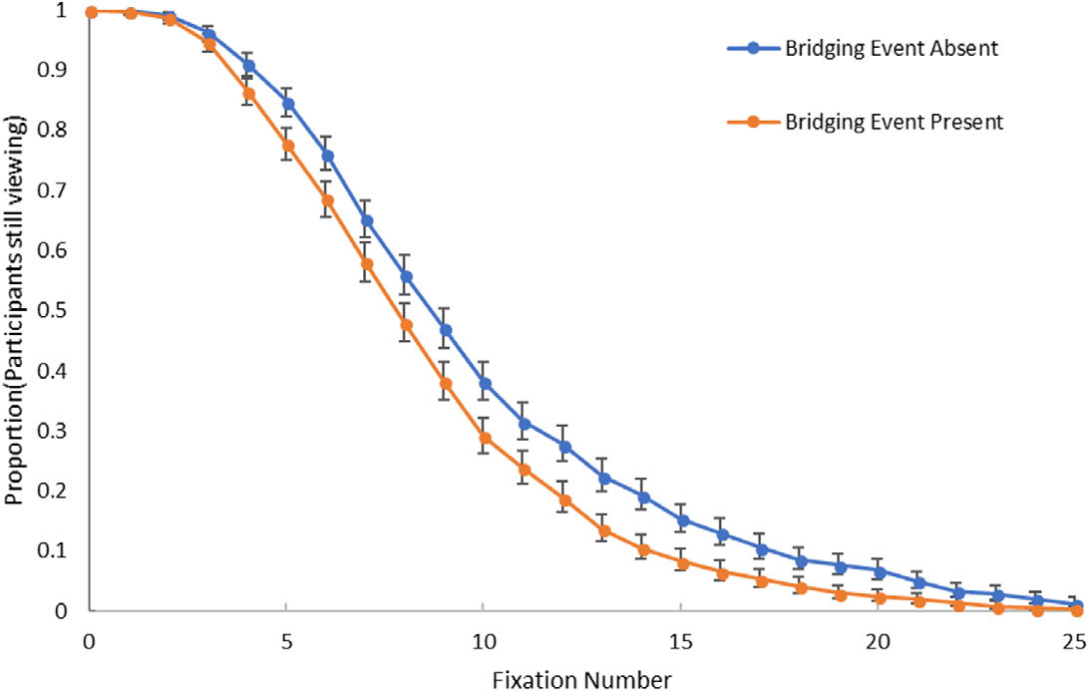
Survival analysis figure showing the proportion of participants still viewing the end‐state image for each ordinal fixation number as a function of bridging event presence (error bars are 1 *SE*).

The omnibus survival analysis showed that bridging event presence did create a divergence point (χ^2^(1) = 33.37, *p* < .001). As shown in Fig. 6, the participants on event‐absent trials appear to have been significantly more likely to continue viewing the end‐state image by approximately the fifth fixation. Since the omnibus survival analysis showed an effect, the three divergence point analyses were run. Both the overall and the confidence interval DPAs showed the divergence point was indeed at the fifth fixation, with the confidence interval analysis showing a lower bound of four fixations and an upper bound of five fixations. Thus, these three analyses agree on the same divergence point.^2^ Based on the average fixation duration given the above mean fixation duration (218 ms), a divergence point at five fixations indicates that it takes participants approximately 1 s (1,090 ms) to identify a break in narrative coherence.

**Figure 7:**
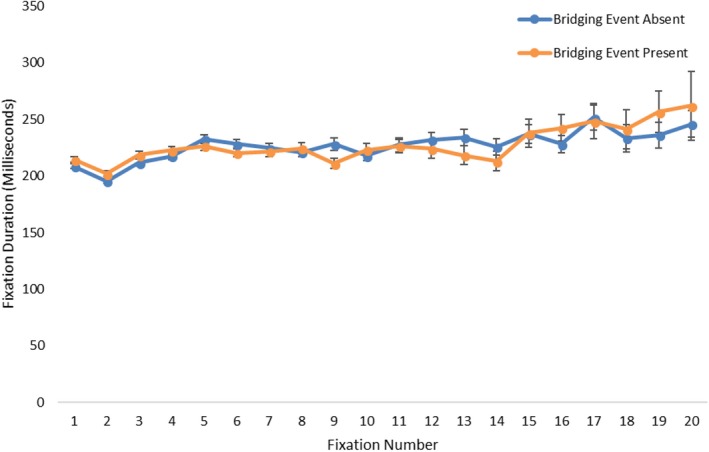
Mean fixation durations (ms) as a function of ordinal fixation number and bridging event presence (error bars are 1 *SE*).

Previous work has found that at the divergence point there may also be a targeted fixation duration effect (Carroll et al., 1992), which the overall fixation duration analysis above may not have had the sensitivity to pick up on. However, Fig. 7 shows that in the study fixation durations were fairly similar across all fixations. Thus, this is more evidence that bridging event presence was not influencing fixation durations, even at the more fine‐grained fixation‐by‐fixation level, nor at the point at which participants identified a break in coherence.

Thus, the eye movement data support the visual search hypothesis but not the computational load hypothesis. When participants perceived a gap in coherence while viewing end‐state images, there was a nearly 20% increase in the number of fixations made, but no increase in the fixation durations. This increase in the number of fixations began on the fifth fixation, indicating that it took approximately five fixations for participants to identify a break in narrative coherence. From a scene viewing perspective, the increase in the number of fixations may have been due to participants searching the end‐state image for clues to support inferring the missing bridging events. Interestingly, if the visual search hypothesis is true, then it suggests the following more refined hypothesis: When the bridging event is absent, the extra fixations should preferentially go to informative regions for generating the bridging inference. We call this the *informativeness* hypothesis.

## 3 Experiment 2: Informativeness of image regions for generating target bridging inferences

The *informativeness hypothesis* suggests that when viewers of a visual narrative are faced with a missing bridging event, they may search the end‐state image for informative cues to generate the bridging inference. There is a long history of using the construct of *informativeness* in explaining attentional selection in scenes, with more informative regions, variably defined across studies, often being more likely to be fixated, or to be fixated for longer (Antes, 1974; Antes & Penland, 1981; Henderson, Brockmole, Castelhano, & Mack, 2007; Henderson & Hollingworth, 1998; Hollingworth & Henderson, 2000). Here, we define a particular type of informativeness, *inferential informativeness*, as being relevant to the specific task of generating a bridging inference. In this sense, it is similar to the construct of *task relevance*, which has also been shown to strongly influence eye movements (DeAngelus & Pelz, 2009; Henderson et al., 2007; Howard et al., 2011; Yarbus, 1967). However, participants’ only explicit task in the eye‐tracking experiment was to view each picture story and then write a brief summary of it. Thus, in contrast to previous studies showing effects of task relevance on eye movements, in this case, the task of generating a bridging inference was completely implicit and was but one theorized subtask in creating a coherent event model for the picture story being viewed, which in the end, enabled participants to write a brief summary of it.

This raises the question, how can one determine which image regions are most informative for generating each bridging inference? The approach we took to answering this question, similar to that of Antes (1974), was to pose it to naïve viewers. Participants were presented with a full picture story, then shown a target episode within that story, and asked to identify the elements of the End‐state image they thought were informative for inferring the missing Bridging event in the bridging event‐absent condition. Our research question was to what degree would naïve viewers’ inferential‐informativeness ratings be related to other participants’ eye movements on the end‐state images?

### 3.1 Methods

#### 3.1.1 Participants

Forty‐two participants (15 women, 27 men) from the Kansas State University undergraduate participant pool participated in Experiment 2 for course credit and were included in analyses. Five participants were dropped for not following the instructions for one or more of the experiment sections (e.g., not labeling items they clicked, or clicking on the same image location multiple times). All participants that completed each section of the experiment as per the instructions were included in the analyses.

#### 3.1.2 Materials

The materials used in Experiment 2 were the same as those in Experiment 1. However, they were presented in a different format, discussed below in the Design and Procedure sections.

#### 3.1.3 Apparatus

Experiment 2 was programmed in Qualtrics, data were collected online, and participants completed it on their personal computers. As such, the technical specifications for the presentation of the picture stories varied between participants.

#### 3.1.4 Design

There were no manipulations in the informative region experiment. Participants were presented the six Boy, Dog, Frog picture stories in full (i.e., without any images missing). To avoid an ordering effect for stories 1–6, the order in which the six stories appeared was randomized across participants.

#### 3.1.5 Procedure

In general, the procedure in Experiment 2 was to tell participants everything about the previous eye tracking experiment that was relevant to their task from the beginning of the experiment and why we were interested in their data. Specifically, participants were told about the manipulation of bridging event presence in the previous eye tracking experiment, and that participants in that eye tracking experiment did not know about the manipulation. Participants’ task was to “indicate features of images you think are important for comprehending a picture story” in the bridging event‐absent condition.

For each story, participants first viewed the entire story without any images missing. After viewing each story, they completed the inference informativeness ratings. To do this, they were presented with each target episode individually. For example, they would be simultaneously presented with both the three‐image and two‐image sequences as shown in Fig. 2, to show what participants in the bridging event‐present and ‐absent conditions saw. In this figure, the bridging event was highlighted, and the action that occurred was named (for Fig. 2 this was “Push Off”). Just below the figure showing the full target episode, there was a large image of the end‐state for that target episode. On this end‐state image, participants clicked the area(s) they thought would be most helpful in making the inference about the missing bridging event for participants in the bridging event‐absent condition for that target episode. After clicking the image areas relevant for making the inference, participants used a textbox to give labels to the locations they clicked. They then followed this same procedure for the rest of the target episodes for the story they saw and then continued on to the next story.

#### 3.1.6 Analysis

The scene region informativeness analysis was designed to test if participants in the bridging event‐absent condition made more fixations to regions that were informative for making the inference. This was done by comparing the correlation values for the inferential‐informativeness click heat maps and the fixation heat maps from the Eye‐tracking Experiment.

**Figure 8:**
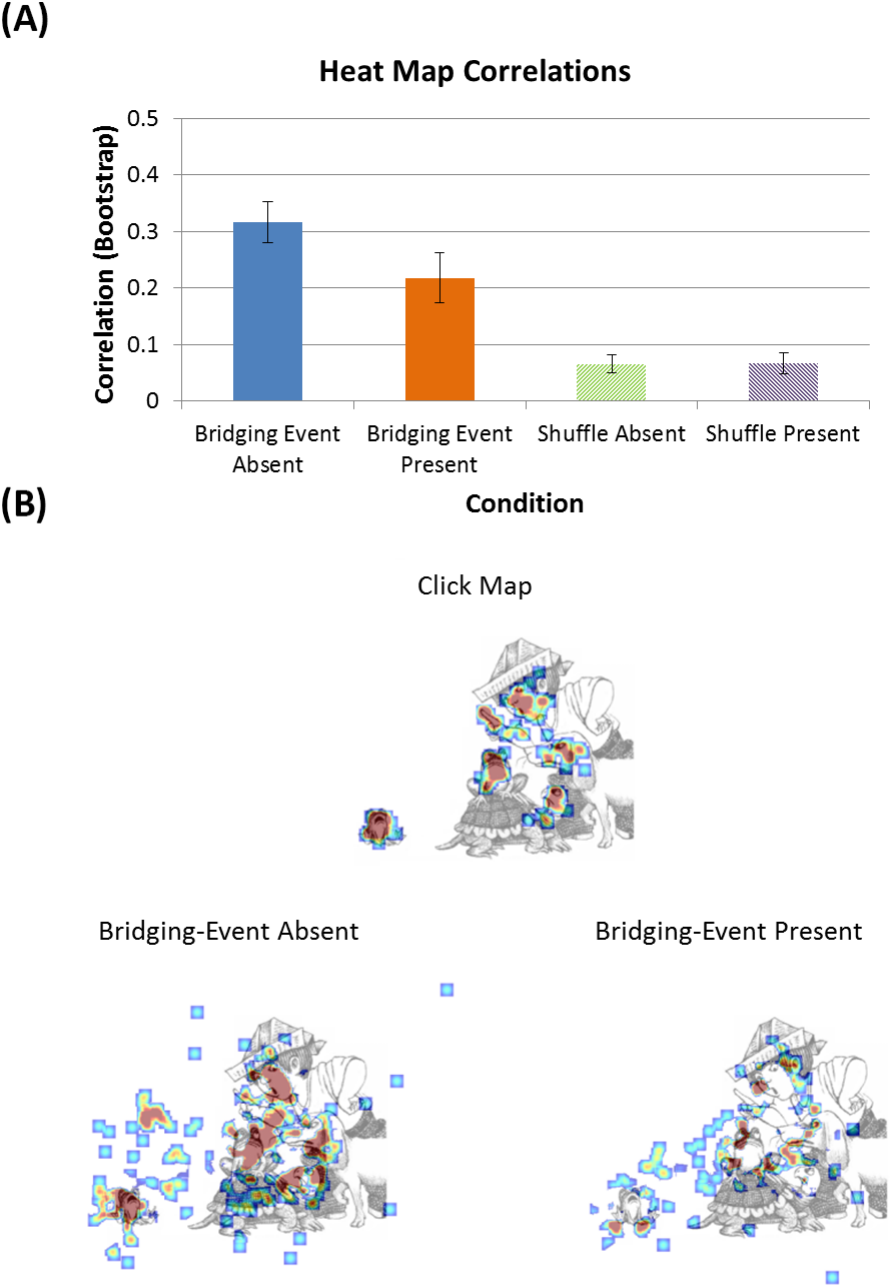
(A) Bootstrapped (1,000 iterations) correlations of bridging event‐absent (blue) and ‐present (orange) unique fixation difference maps with inferential‐informativeness click maps (correlations averaged across all images for each condition). Error bars are 95% confidence intervals. Shuffled baseline correlations for bridging event‐absent (green striped) and ‐present (purple striped) were for unique fixation difference maps paired with randomly selected click maps. (B) Example end‐state image heat maps. On top is the inferential‐informativeness click map that the unique fixation difference heat maps were correlated with. The bottom left figure shows the unique fixation difference heat map in the bridging event‐absent condition. The bottom‐right figure shows the opposite, fixation locations unique to bridging event‐present participants.

To compare the inferential‐informativeness click heat maps with the fixation heat maps, the data were processed and analyzed in MATLAB, and visualizations were done, using the Image Processing Toolbox. First, the fixation and click maps were transformed to have the same resolution (800 × 600). There was one click map and heat map per image and condition (e.g., for the End‐state image in Fig. 2 [and Fig. 8B] there was a click map for all participants that did the click experiment [Experiment 2], a fixation heat map for all participants in the bridging event‐present condition in the eye tracking experiment [Experiment 1], and a fixation heat map for the bridging event‐absent condition [Experiment 1]). These maps had a value of 1 at the pixel location a participant fixated or clicked. Next, a Gaussian filter was run on the click and fixation maps, which smoothed the fixation and click locations over 1° of visual angle. This Gaussian smoothing procedure created the heat maps. With the smoothed heat maps, the next step was to create unique fixation heat maps for the bridging event‐present and bridging event‐absent conditions, and then unique fixation location maps based on the differences between them. To do this, for each end‐state image, the bridging event‐present heat map was subtracted from the bridging event‐absent map, and vice versa. With these unique fixation density difference heat maps, bootstrap correlations (1,000 iterations) were run for each end‐state image. Specifically, the inferential‐informativeness click map for a single end‐state image was correlated with the bridging event‐absent unique fixation difference map and the bridging event‐present unique fixation difference map for the same image. This was done for each end‐state image. The correlations were run for the entire difference heat map, meaning that each correlation quantified the relationship between the specified click and fixation difference heat maps on a pixel‐by‐pixel basis (e.g., the value for click map pixel 1, 1 [top left corner] was compared to bridging event‐present fixation difference heat map pixel 1, 1, then pixel 1, 2, and this continued until each pixel location was run). The bootstrapping procedure used the bootstrap function in MATLAB (bootstrp), which samples with replacement to calculate the specified statistic (correlation in this case) for the specified number of iterations (1,000). The bootstrapping procedure was used to obtain a robust estimate of the correlation value and to calculate 95% confidence intervals for the correlation values, which allowed for comparison between the bridging event‐absent and ‐present conditions. Additionally, a shuffled baseline was calculated for each image, which should show the expected average correlation between unique fixation difference and inferential‐informativeness click maps for the Boy, Dog, Frog picture stories. The shuffled baseline was created by randomly pairing inferential‐informativeness click maps to unique fixation difference maps for each image, and then running the bootstrap correlation. This was done by randomly pairing five click maps with each unique fixation difference map. By comparing the true inferential‐informativeness click map to unique fixation difference map correlations with the shuffled baseline correlations, we can determine the degree to which any correlations seen are due to the bridging event presence manipulation, and not a result of random patterns of fixation locations that may occur. For example, it is well known that when viewing pictures on a computer screen, there is a strong bias to fixate in the center (Tatler, 2007), which would inflate the correlation values regardless of condition. The shuffled baseline is a way of accounting for such randomly generated correlations.

Statistical comparisons between the bridging event presence conditions were based on the 95% confidence intervals calculated using the bootstrap procedure. If the 95% confidence intervals overlapped, the difference was not statistically significant (i.e., *p* > .05), but if the confidence intervals did not overlap, the difference was statistically significant (i.e., *p* < .05).

### 3.2 Results

The results showed that when the bridging event was absent (thus a bridging inference was needed to maintain coherence), participants made more fixations to the informative regions of the end‐state scene than when the bridging event was present (thus no bridging inference was needed) (Fig. 8). The average correlation value for the bridging event absent trials was *r* = .32 (95% CI [.28, .35]), and for bridging event present it was .22 (95% CI [.17, .26]). Importantly, shuffled baselines were also run to identify the random baseline correlation level for the images used in the study. The correlations for the shuffled baseline for both the bridging event‐absent (*r* = .07, 95% CI [.05, .08]) and ‐present (*r* = .07, 95% CI [.05, .08]) conditions were the same out to two decimal places, and well below the actual inferential‐informativeness click map to unique fixation difference heat map correlations. Based on the low shuffled baseline values for both the event‐present and ‐absent conditions, it is clear that the correlation results are *not* due to fixations randomly falling in the inference informative regions *nor* are the correlations an artifact of the images chosen (e.g., due to a center bias). The fact that the event‐absent and ‐present shuffled baselines have virtually identical correlations and 95% confidence intervals also indicates that the effect for the experimental conditions is not driven simply by the extra fixations in the bridging event‐absent condition increasing the likelihood fixations would fall on inference informative regions. If this were the case, then one would expect that the shuffled baseline for the bridging event‐absent condition would have a higher correlation than the shuffled baseline for the bridging event‐present condition. However, that was not the case, thus ruling out that explanation of the higher correlation in the actual event‐absent condition.

Taken together, these results support the inferential‐informativeness hypothesis. The manipulation of bridging event presence, which determines whether viewers need to draw a bridging inference, affects what scene information viewers fixate, namely their attentional selection. When participants need to make an inference, they fixate regions of the scene that are helpful in drawing the bridging inference.

Experiment 1 analyses showed that participants made more fixations in the bridging event‐absent condition, beginning on the fifth fixation, which suggested they were using those extra fixations to search for information that would allow them to generate the bridging inference necessary to maintain a coherent event model in working memory. The informativeness analysis tested this by having participants identify scene regions important for generating the inferences, and then comparing the eye movements of participants to the inferentially informative regions. In support of the informativeness hypothesis, for bridging event absent trials, which require an inference to maintain event coherence, participants were more likely to fixate scene regions identified as informative for making the inference.

## 4 General discussion

When people view and comprehend a visual narrative, they must necessarily coordinate processes involved in both visual scene perception and event model construction. However, studies of visual narrative comprehension have been hampered by the fact that there has been no theoretical framework that explains this coordination. SPECT fills this void. SPECT argues that back‐end processes involved in creating the current event model are coordinated with the front‐end processes of information acquisition and attentional selection. This study tested key components of SPECT. Two novel competing hypotheses tested why there are longer viewing times on pictures that require a causal bridging inference (Cohn & Wittenberg, 2015; Magliano, Kopp, et al., 2016; Magliano, Larson, et al., 2016). The computational load hypothesis was that longer viewing times would be caused by having longer fixation durations, based on needing additional processing time in WM. The competing visual search hypothesis was that longer viewing times would be caused by making more fixations, based on needing to gather additional information to support either generating the inference, or validating an inference the viewer had already generated. The results of our study provided clear evidence in favor of the visual search hypothesis, with participants making more fixations on the end‐state image when generating an inference (Experiment 1; Fig. 5) beginning around the fifth fixation (Experiment 1; Fig. 6), and fixating inferentially informative regions with those extra fixations (Experiment 2; Fig. 8A)—supporting the informativeness hypothesis.

This study tested of a novel hypothesis of SPECT that, while viewing a visual narrative, back‐end processes involved in creating the current event model in working memory should affect front‐end processes during individual eye fixations, in this case, attentional selection (see also Magliano et al., 2013, in press). More specifically, the hypothesis was that, while viewing a visual narrative, the back‐end process of mapping in‐coming information to the current event model, which includes the subprocess of generating bridging inferences to maintain coherence, would influence the front‐end process of attentional selection during fixations, namely where viewers would look. If the products of the front‐end process of information extraction do not allow the back‐end process of mapping in‐coming information to maintain a coherent event model, this provides a signal to the front‐end process of attentional selection to search the current picture (or prior story content in working memory or long‐term memory when available) for content that can support the bridging inference. If this process fails, according to SPECT, the back‐end process of shifting to create a new event model is initiated.

What signals the viewers during the mapping process that they need to draw a bridging inference? According to the event‐indexing model, in text and film, people track key components of the narrative for changes or breaks in coherence (Magliano, Taylor, & Kim, 2005; Magliano, Zwaan, & Graesser, 1999; Zacks et al., 2009; Zwaan & Radvansky, 1998). We assume this coherence monitoring process is performed by mechanisms similar to those described by Kintsch's (1988, 1998) Construction‐Integration (CI) model of comprehension (McNamara & Magliano, 2009). According to the CI model, coherence is achieved in two stages. The first stage is knowledge *activation*, and the second stage is *integration* of that activated knowledge into a coherent situation model (or *event model* here). Integration is assumed to be driven by constraint satisfaction due to spreading activation (Kintsch, 1988). SPECT therefore assumes that the products of front‐end information extraction (recognition of scene gist, objects, and actions) lead to the activation of knowledge from the current event model and relevant background knowledge in long‐term semantic memory. When two images in a visual narrative have considerable overlap in semantic features, the integration phase within the mapping process is completed relatively quickly and the event model in working memory reflects a coherent sequence of actions. This provides a signal that no additional mapping processes are needed, and the viewer moves on to the next panel/picture in the visual narrative. Conversely, when there is a semantic feature gap in the narrative, the integration phase of the mapping process takes longer because the front‐end process of information extraction has produced relatively more disparately associated knowledge during the activation phase. Thus, a less associated network, which is less coherent, is established at the end of the integration phase of the mapping process. This signals the need for additional constructive processes to maintain coherence to complete the mapping process (e.g., Long & Lea, 2005), namely generating a bridging inference.

These constructive processes have been characterized as explanatory in nature, in that comprehenders have a drive to understand *why* events occur in narratives (Graesser et al., 1994). Thus, while processing a visual narrative, having a less coherent network at the end of the integration phase of mapping produces an error signal. That error signal guides the front‐end attentional selection process to search the image for information that, when rapidly extracted, can activate knowledge that is useful for generating a bridging inference that achieves an acceptable level of coherence. The survival analysis in Experiment 1 showed that it took readers of picture stories approximately five fixations (1 s) to complete the construction and integration phases and identify the break in coherence. Carroll et al. (1992) showed a similar time course (7 fixations) for understanding a joke when participants were first presented with the text of a The Far Side comic, and then the image meant to accompany the text. These time course results are very interesting for SPECT. Larson (2012) showed that while you get the gist of the scene location (first the superordinate level, e.g., indoor vs. outdoor, then the basic level, e.g., kitchen vs. office) within the first fixation, participants required an extra fixation to recognize the key basic level action in a scene (e.g., cooking vs. washing dishes). Based on this, it makes sense that it would take a few extra fixations beyond the first two fixations that acquire scene gist and basic level actions to (a) identify that there was a break in coherence, and (b) to begin efforts to draw a bridging inference to fill that gap.

Research on the time course of inferences in text processing suggests the range of completion from 750–1,500 ms after the final word of a sentence is processed (Magliano et al., 1993; Till, Mross, & Kintsch, 1988), which is a similar time frame to the eye movement results. While it is interesting that we found evidence of eye movements associated with bridging inferences occurring during this temporal range in visual narratives, we caution over‐interpretation. This is in part because text and visual narratives require different front‐end processes to be completed before inferences can occur (Magliano et al., 2013). Moreover, some processes that support comprehension likely occur in parallel but are asynchronous (O'Brien & Cook, 2016), and therefore it is difficult to make direct comparisons between the time course of inferences for text and visual narratives. Nonetheless, we find the parallels between this study and research on texts intriguing and warranting further investigation. Physiological measures that may provide converging evidence of inference generation in picture stories are the ERP signals that correspond with information updating in the mental model and reactivation of narrative information no longer in working memory (Cohn & Kutas, 2015).

It is tempting to make comparisons of the time course of processing bridging inferences based on our eye movement measures and previous studies investigating the same sort of time course of inference generation based on ERPs (e.g., Cohn & Kutas, 2015). However, it is difficult to make direct comparisons of our eye movement‐based results with such ERP results due to the differences in the viewing conditions and the stimuli used. For example, the Cohn and Kutas (2015) ERP study restricted eye movements, whereas our study allowed free eye movements. Their study also had much simpler stimuli (based on Peanuts comics) than our study. Thus, making eye movements was likely less important in their study (e.g., the average number of fixations in Peanuts comics varied from 5.4 to 5.6 in Foulsham, Wybrow, & Cohn, 2016) than in ours (e.g., the average number of fixations in our study varied from 9 to 11). Nevertheless, Cohn and Kutas (2015) found evidence of noticing a gap in coherence (i.e., “prediction error”) based on the P600 ERP after anywhere from 400 to 900 ms following onset of the final image (similar to our end‐state image). In contrast, we found evidence of having noticed a gap in coherence after five fixations, which we estimate to have been after roughly 1,090 ms (i.e., 5 × the mean fixation duration of 218 ms). This raises an interesting question, namely, when would our participants have produced a P600? Assumedly, it would have begun prior to the differential movements of their eyes. Further research will be needed to answer such questions.^3^

A key question that can be asked about our results is regarding the direction of causality. Correlation does not imply causation, and the relationship we have shown between inferential informativeness click maps and fixation heat maps is just that. So, one may rightly ask, what is the direction of causality? Does back‐end inference generation cause changes in attentional selection, or does front‐end attentional selection cause changes in inference generation, or does a third factor cause both to vary? To answer this, we point to the fact that, unlike correlations, experiments do allow one to make claims about the direction of causality. Specifically, we know that our experimental manipulation of narrative coherence (through presence/absence of the bridging event) causes an increase in inference generation, as shown by both think‐aloud data (increased mention of the unseen bridging events: Magliano, Larson, et al., 2016) and recognition memory data (increased false alarms to unseen bridging events: Magliano, Kopp, et al., 2016). Furthermore, that same coherence manipulation also causes viewers to make more fixations (Experiment 1). Therefore, the causal attribute is the processing of coherence relations across images viewed at different times, which must occur in WM. This is consistent with back‐end coherence processing, which results in inference generation (in SPECT's event model, in the mapping stage) causing changes in front‐end attentional selection. But can our results be explained just as well by the reverse direction of causality? In that direction, the causal attribute is the front‐end, stimulus saliency in the End‐state image, which draws attention, producing changes in eye fixations, which cause changes in inference generation. We can reject this causal explanation of our results because the End‐state image, and its accompanying stimulus saliency, is exactly the same in both coherence conditions, and should therefore produce no differences in eye movements. Thus, while the relationship we have shown between inferential informativeness and fixation location is correlational, our results show that coherence monitoring has a causal effect on visual attentional selection, which cannot be explained by visual saliency.

### 4.1 What is the search template?

If we posit that viewers are searching for inferentially‐relevant information, just what sort of information is used in the visual search template during the construction and integration phases? During the construction phase, search is likely to go to narrative/scene consistent regions (e.g., to locations where agents are predicted to be) (Eckstein, Drescher, & Shimozaki, 2006; Neider & Zelinsky, 2006). When agents are missing, or agents’ goal‐states have changed, this creates a break in coherence during the integration phase and is likely what leads to the search for inferentially relevant information. What inferentially relevant information drives the search during the construction phase?

Note that in the traditional visual search literature, the search template is often very explicitly known (e.g., search for the conjunction of red + vertical among red and green items that are vertically or horizontally oriented). However, more recent studies of more natural visual search have shown that people can also very successfully search for categorically defined targets (Maxfield, Stalder, & Zelinsky, 2014; Zelinsky, Adeli, Peng, & Samaras, 2013), such as a “chair,” which necessarily have variable perceptual features (e.g., a wooden dining chair, vs. an aluminum and plastic deck chair, etc.). Other studies have shown that viewers can successfully search for even more abstract categories such as “suspicious behavior” in closed‐circuit TV surveillance video (Howard et al., 2011), which again would fit with a wide variety of complex perceptual features, but all of which are meaningfully connected. Visual search for information relevant to drawing an inference would likely be as abstract or more so.

Two possible types of search templates could be used to guide visual search for inferentially‐relevant information: (a) key information that is generally needed in an event model (e.g., agents or goals) but is missing, and (b) hypothesized critical information, based on candidate bridging inferences already generated through the activation and integration processes. The first type of search template is more similar to a bottom–up comprehension process (see Myers et al., 2000), in which the overlap between the event model and current scene information lead to an inference through spreading activation (Myers & O'Brien, 1998). Conversely, generating inferences and searching for confirmatory information is an active, strategy‐based, top–down comprehension process (van Dijk & Kintsch, 1983; Myers et al., 2000). Importantly, both of these search processes could occur, with the initial search for missing event model information leading to the generation of candidate inferences that then leads to new search targets. The extent to which each occurs would likely depend on the information available in the scene and the strength (i.e., probability) of the inference that can be drawn from it.

To illustrate, consider the example shown in Fig. 3, which illustrates the missing bridging event, “kicked off” [big frog, little frog, turtle's back], and Fig. 8B, which shows the details judged to be inferentially relevant in the end‐state image, and the areas of that image selectively fixated in the bridging event‐absent condition. We assume that viewers in the event‐absent condition begin with an event model for the beginning‐state image they just looked at (e.g., “boy and pets going for a walk, big and little frog riding on turtle's back, big frog angry at little frog”). Then, on seeing the end‐state image, viewers would likely initially need to search to identify the states of the agents in the scene. After identifying those, a comparison to their current event model (from the beginning state image) shows two new events have occurred, namely (a) little frog sitting on the ground crying, and (b) boy, dog, and turtle angry at big frog who is still on the turtle. Because these two new events differ from the current event model (i.e., “walking” + “riding”), the viewer would need to generate a causal bridging inference to explain the new events to maintain the coherence of the current event model (Graesser et al., 1994). Based on the information available in the scene, this inference may be drawn and seem likely enough that the reader continues. However, if the reader is less certain in her inference, she may also additionally search for confirmatory evidence for her inference. Fig. 8B shows that the inferentially relevant areas of the image were primarily judged to be the faces of each of the characters/entities (boy, dog, turtle, big frog, and little frog), and these same areas were also preferentially fixated by viewers in the event‐absent condition. The entities’ facial expressions and their lines of gaze are consistent with the above‐noted two new events (i.e., (a) sad little frog on ground, and (b) others angry at big frog), but the question for the viewer is “Why” (Graesser & Clark, 1985; Graesser & McMahen, 1993)? As noted above, the viewer's current event model likely included the fact that the big frog was angry at the little frog (see Fig. 3, beginning‐state). Thus, viewers might infer that the big frog likely caused the little frog's crying on the ground. But how could the little frog have ended up there? A plausible answer based on knowledge from long‐term semantic memory of aggressive actions is that “kicking” or “shoving” someone can make them fall to the ground and cry. Together, this would produce the inference that the big frog kicked/shoved the little frog off the turtle onto the ground. To confirm this inference, the viewers may then have (re)fixated one or more of the entities’ faces, producing the added fixations there. The fact that the viewers in the bridging event‐present condition looked less at each of the entities’ faces suggests that they required less information from those faces to understand the two new events. Assumedly, this was because the new events were more predictable given that their current event model included the bridging event (i.e., big frog kicked little frog off the turtle). More generally, viewers may search the image to test their generated inference, the specifics of which are captured by viewers’ intuitions as to the inferential‐relevance of information in the image.

The above raises the question, why visually search for inference‐relevant information in the image rather than simply spend extra computational resources in working memory? The short answer is that it may be “cheaper” to use eye movements than to integrate large amounts of information into the event model in WM (Ballard, Hayhoe, & Pelz, 1995; Hayhoe, Bensinger, & Ballard, 1998). Agent goal‐state information is likely needed to make an inference during the integration phase. This process likely requires the binding of multiple agents’ or entities’ goal‐state information within the scene to identify how they have changed from what is in the current event model (e.g., the above example of the little frog going from riding on the turtle to being on the ground crying). Once the viewer has generated a likely candidate inference, confirming it can either be done within WM, or by directly targeting the specific entities deemed to be more inferentially relevant. Note that only the last 3–4 most recently fixated objects are highly active in WM (Hollingworth, 2004; Zelinsky & Loschky, 2005), with items fixated earlier than that being at a significantly lower, but above‐chance level. Thus, as more agents/entities are fixated, some previously fixated items may no longer be highly active in WM. Therefore, to refresh that information, viewers may choose to refixate certain items (Zelinsky, Loschky, & Dickinson, 2011). Furthermore, since binding object information is cognitively demanding (Simons, 1996), not all agent/entity information is likely integrated during a single fixation. Studies have shown that in visual tasks, viewers generally prefer to refixate items to extract new information from them, rather than to depend on WM, even when the information is well within their working memory limits (Ballard et al., 1995; Hayhoe et al., 1998). In other words, since scene information remains available, only the currently goal‐relevant information is integrated into the event model.

### 4.2 Modality specific processing

There has been speculation about the extent to which the processes that support comprehension of text and visual narratives are modality‐specific or ‐independent (Gernsbacher, 1990; Kintsch, 1998; Magliano et al., 2013; Magliano, Larson, et al., 2016). While there is evidence that some aspects of narrative comprehension are similar across the text and visual narrative modalities (Baggett, 1979, 1989; Cohn & Kutas, 2015, 2017; Magliano et al., 2013), due to the differences in stimulus type, the front‐end (perceptual) processes supporting them are almost certainly unique (Loughlin et al., 2015; Sadoski & Paivio, 2004). Bridging inferences are a candidate for a process that is important to comprehension for all modalities because it is fundamental to event model construction (Graesser et al., 1994). Pictures in visual narratives afford visual search when one recognizes that there is a break in the causal cohesion. An interesting question is the degree to which text affords something similar to a visual search. An argument can be made that the regressive eye movements that readers make when faced with a clear break in coherence (Calvo et al., 2001; Poynor & Morris, 2003) are similar in nature. Such eye movements are done to reanalyze previously read text in an effort to find information that can lessen the perceived lack of coherence.^4^ However, based on a probe question methodology with text, readers often rely more on knowledge activation to support the computation of causal bridging inferences (Singer & Halldorson, 1996), with most such relatively trouble‐free inference generation resulting in longer gaze durations, but not regressive saccades to search for information (Ehrlich & Rayner, 1983; Myers et al., 2000; O'Brien et al., 1988).

It is likely that there may be other aspects of back‐end event model construction that are common across modalities, but they are supported by front‐end processes that are unique across modalities (Just & Carpenter, 1980; Loughlin et al., 2015). This study makes a strong case that future research is needed to address this issue (see also Cohn & Kutas, 2015, 2017). It involved presenting pictures one at a time, which is consistent with the format for visual narratives (e.g., children's picture stories). However, comics consist of multiple panels on one page and afford visual search of prior panels, which was not possible in this study (viewers could not go back to review previous images in a story). There is some evidence indicating that there are regressive eye movements to prior pictures in the context of a comics layout (Foulsham et al., 2016). However, without the use of layout in this study, the current work could not explore the extent to which inference generation was associated with regressive eye movements. As such, future research should explore how eye movements support bridging inferences in a comics layout.

In conclusion, this study presents novel work on eye movements and comprehension in visual narratives, and it points toward a need for future work testing visual search while reading text versus picture stories. The main contribution of the current work is that it is an early step in testing the relationship between scene perception and event comprehension as proposed by SPECT, and the time course of this relationship. The central finding that participants searched for inference‐relevant information when the visual narrative they were reading induced bridging inference generation is a unique contribution to our understanding of how visual narratives are comprehended, and it makes the need for a theory such as SPECT clear.

## Funding

This research was partially funded by the Center for the Interdisciplinary Studies of Language and Literacy (JM).

## Author contributions

Conceived and designed the experiments: LCL, JPH, and JPM. Performed the experiments: JPH. Analyzed the data: JPH, LCL, and JPM. Contributed reagents/materials/analysis tools: JPM and LCL. Wrote the paper: JPH, LCL, and JPM.

## Notes

1 In addition to the general viewing time effects being replicated from Magliano, Larson, et al. (2016), the mean viewing times were also very similar: Bridging event‐absent: *M* = 2,738 ms (compared to *M* = 2,788 in this study); Bridging event present: *M* = 2,556 ms (compared to *M* = 2,369 in this study).

2 The third divergence point analysis (individual participants’ divergence points) returned a divergence point at the first fixation. A divergence point at the first fixation would mean that a person only needs one fixation to get all the information she needs to identify whether there was a break in narrative coherence. However, previous research has shown that while the gist of a scene (e.g., that a location is a kitchen vs. an office) can be extracted within a single fixation, two fixations are needed to extract basic‐level action information (e.g., that a person is cooking vs. washing the dishes) (Larson, 2012). Since people need more than a single fixation to acquire basic level action information in a scene, and three of the four analyses converged on a value of five fixations, the individual participant divergence point of 1 is likely to be spurious. Additionally, at the statistical level, the analysis did not identify divergence points for two of the participants, which could have produced the low divergence point estimate. When the participants without a divergence point are removed, the average divergence point calculated from each participant's divergence point median was 6.29 fixations (lower CI = 6.25; upper CI = 8.38). This is consistent with findings that the individual difference convergence point estimates tend to be biased toward slightly higher values than the original or confidence interval estimates (Reingold & Sheridan, 2014).

3 However, there are non‐trivial analytical issues to be solved. Chief among them is how to handle analysis of EEG data averaged across multiple participants across multiple fixations. Current state‐of‐the‐art research that combines analysis of eye movements and EEG (Henderson, Luke, Schmidt, & Richards, 2013) tends to time lock ERPs to the start of a fixation, but it does not take account of the ordinal number of fixations in sequence (such as our 9–11 fixations on the End‐State image). Additionally, the ERP components of interest (e.g., P600) have a time course longer than a normal single fixation (218 ms in our study).

4 This raises the question, does this study constitute a case of perceived incoherence? In general, the bridging events were more inferable than not (59% of the inferable actions in the bridging event‐absent condition were mentioned by participants in a think‐aloud experiment in Magliano, Larson, et al., 2016). Furthermore, most, but not all, of the bridging events were consistent with the protagonist's goals. Nevertheless, we assume that in the bridging event‐absent condition, the viewer was always faced with a break in coherence that required her to generate a bridging inference.
